# Grape seed and skin extract, a potential prebiotic with anti-obesity effect through gut microbiota modulation

**DOI:** 10.1186/s13099-022-00505-0

**Published:** 2022-07-06

**Authors:** Mohamed Mokrani, Kamel Charradi, Ferid Limam, Ezzedine Aouani, Maria C. Urdaci

**Affiliations:** 1 Bordeaux Sciences Agro, CNRS, University of Bordeaux, UMR 5248, CBMN, Interactions Bactéries Probiotiques Hôte, 1, Cours du Général de Gaulle, 33175 Gradignan Cedex, France; 2 Laboratory of Bioactive Substances, Center of Biotechnology of Borj Cedria, BP901, 2050 Hammam Lif, Tunisia

**Keywords:** Obesity, Grape seed and skin extract (GSSE), Orlistat, Gut microbiota dysbiosis

## Abstract

**Background:**

Obesity is a worldwide health problem and a significant risk factor for diabetes and cardiovascular diseases. Gut microbiota (GM) plays an essential role in obesity, and prebiotics such as polyphenols could be one way to improve microbial dysbiosis-induced obesity.

**Objective:**

This study was designed to assess the effectiveness of grape seed and skin extract (GSSE), and/or orlistat on obese rats fed with high fat diet by targeting GM modulations. The impact of treatments was also studied in non-obese rats.

**Material and methods:**

Rats were rendered obese or kept with a standard diet for three months. Then they were treated either with GSSE or orlistat or with the combined treatment (GSOR) during three months and then sacrificed. Adipose tissues, blood and faeces were collected and analyzed.

**Results:**

In obese rats and to a lesser extent in non-obese rats, treatments decreased the weight of various adipose tissues and the serum levels of cholesterol, LDL, triglycerides, lipase, and CRP and increased HDL and adiponectin. GSOR treatment was even more efficient that orlistat. Obese rats had less GM diversity than non-obese rats and orlistat reduced it even more. However, diversity was restored with GSSE and GSOR treatments. Potential pathogenic *Streptococcus alactolyticus/gallolyticus* species were greatly increased in obese rats and drastically reduced with the treatments, as wells as other potential pathobionts.

**Conclusions:**

GSSE exerts beneficial effects in obese rats and restores, at least partially, the observed dysbiosis. GSOR induced the highest beneficial effect. Moreover, the various treatments could also enhance physiological and GM modifications in non obese rats.

**Supplementary Information:**

The online version contains supplementary material available at 10.1186/s13099-022-00505-0.

## Background

For several decades, most countries worldwide have faced an obesity epidemic, and according to the World Health Organization, more than 1.9 billion adults (older than 18 years) are overweight, and 600 million adults are obese [1]. This problem is reported in western countries and also in developing countries because of the lower cost of obesogenic foods [2]. Additionally, obesity is also associated with the increasing incidence of related metabolic disorders such as type 2 diabetes (T2D), which can raise the risk of cardiovascular disease by twofold [3]. The Middle East and North Africa region carried the highest prevalence of diabetes in 2019 at 12.2%, with a predicted 30% obese by 2030 [4].

Low-grade “metabolic” inflammation has been identified as a contributor to the development of insulin resistance and progression to T2D [5]. Chronic adipose tissue inflammation caused by obesity and T2D can increase inflammatory indicators like cytokines and chemokines linked to metabolic health issues [6, 7].

The evolution of metagenomic tools suggests the strategic use of gut microbiota (GM) as a disease indicator [8]. In fact, GM dysbiosis has been linked to obesity and related pathologies, eg. irritable bowel disease (IBD), and cancer [9–12]. Furthermore, many studies [12, 13] demonstrated that changes in GM composition represent one of the significant factors involved in the development of hepatic dysregulation, low-grade inflammation, and increased permeability in high-fat-diet (HFD) fed mice [13, 14]. *Akkermansia muciniphila* has been identified as a critical component of intestinal microbiota which prevents metabolic disorders such as obesity and T2D [15, 16]. On the other hand, diets rich in fat result in an over-representation of pathogenic lipopolysaccharides (LPS) produced by *Proteobacteria* and subsequent metabolic endotoxemia [17].

A recent study demonstrated that daily low-fermentable fiber supplementation could improve insulin sensitivity following fecal microbial transplantation by differential GM modulation [18]. Consequently, microbiota modifications can be a good approach as a preventive and curative tool [19, 19]. Suitable dietary interventions can alter GM composition by improving GM’s low genetic richness and clinical phenotype [21].

Prebiotic compounds such as fructo-oligosaccharides (FOS) and galacto-oligosaccharides (GOS) were recognized for their ability to exert beneficial effects on host microbiota [22]. For a few years, dietary polyphenols were considered as prebiotics by reshaping the GM balance [23–25]. Grape seed and skin extract (GSSE) is a coproduct of vinification constituted by a complex mixture of polyphenols, mainly proanthocyanidins, flavonoids, non-flavonoids, and stilbenes as resveratrol [26]. GSSE is a potent antioxidant and has been demonstrated to possess anti-inflammatory effects [27]. Moreover, GSSE exerts a positive effect against obesity and other metabolic syndrome pathologies [28, 29]. Orlistat is a reversible gastric and pancreatic lipase inhibitor used in more than 120 countries as an antiobesity drug treatment [30, 31]. However, orlistat marketed as Xenical and Alli [31] could also provoke adverse side effects such as oily stools, diarrhea, abdominal pain, and fecal spotting with a few cases of severe hepatic adverse events [32]. Mahmoudi et al. [28] demonstrated that combining GSSE and orlistat could improve significantly the anti-obesity and anti-lipotoxicity effects of the drug.

Therefore, the objective of this work was to study the capacity of three treatments, GSSE, orlistat and the combination of both, to improve physiological parameters linked to obesity and to study thoroughly their effect on GM modulation, in an HFD model and rats fed a standard diet (SD). Data support the application of GSSE combined with orlistat in obesity treatment and GM modulation.

## Results

### Physiological parameters

**Fig. 1:**
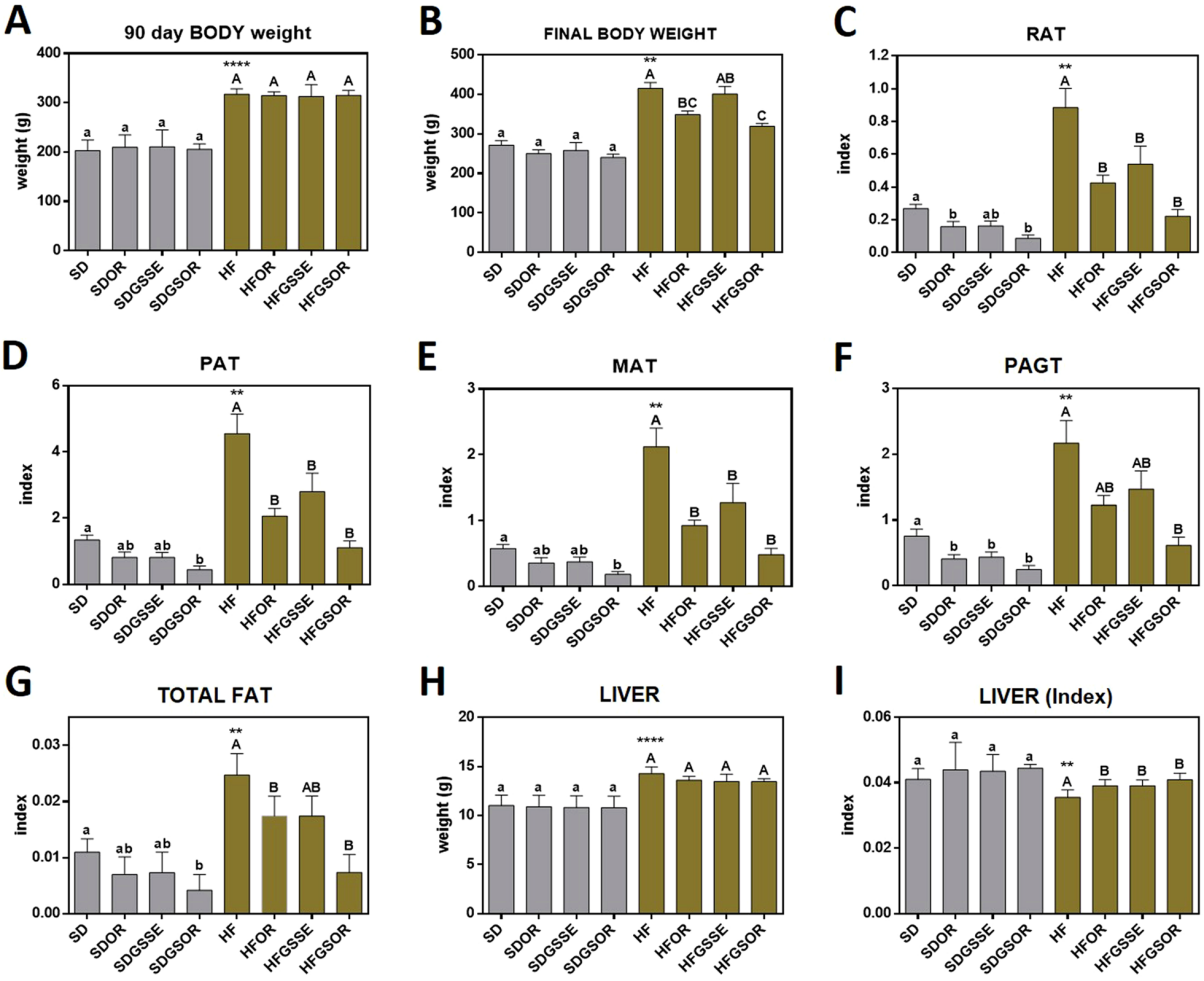
Effect of OR, GSSE, and GSOR on SD (grey) and HF diet (brown)-induced 90 days body weight (**A**) final body weight (**B**) RAT index (**C**), PAT index (**D**), MAT index (**E**), PAGT index (**F**),, fat/final body weight index (**G**), liver weight (**H**) and liver weight index (**I**). Data are expressed as mean ± SEM (n = 6). On top of each bar, lowercase and capital letters indicate significant differences analyzed by two-way ANOVA followed by Tukey’s test (P < 0.05) for vs.SD and vs. HF diet, respectively. The asterisk represents the significant difference analyzed by the parametric t-test (P < 0.05) for SD vs. HF. **** indicates 0.001 < P-value < 0.01; *** indicates 0.001 < P-value < 0.01; ** indicates 0.001 < P-value < 0.01; * 0.01 < P-value < 0.05

After the first three months, animals fed with HFD became obese (Fig. 1A). At this time, rats were treated with GSSE, orlistat (OR), or GSSE + orlistat (GSOR) for three more months according to their corresponding diet. Only obese rats treated with OR or double treatment had reduced weight gain (Fig. 1B), without any effect on food intake (Table 1). In rats fed SD (lean rats), none of these treatments modified their final body weight.

**Table 1:** Effect of GSSE and Orlistat on food and energy intake: data are presented as ± SEM

Parameter	SD	SDOR	SDGSSE	SDGSOR	HF	HFOR	HFGSSE	HFGSOR
Energy intake in the end of treatment (kcal/day)	53.44 ± 4.912	50.6 ± 6.082	52.34 ± 3.568	52.21 ± 3.4	90.5 ± 7.494*	91.51 ± 5.524	91.91 ± 7.778	89.19 ± 6.966
Food intake in the end of treatment (g/day)	20.24 ± 1.86	19.17 ± 2.304	19.83 ± 1.351	19.78 ± 1.288	21.2 ± 1.755	21.43 ± 1.294	21.53 ± 1.821	20.89 ± 1.631

p < 0.05 was considered significant. HFD vs. SD: *p < 0.05;

In HFD animals, the weight of total fat, RAT, PAT, MAT, and PGAT adipose tissues indexes increased significantly compared to SD rats (Fig. 1C–G). Liver weight was significantly higher in HFD rats than in SD rats (Fig. 1H). In SD as well as HFD animals, OR and GSSE affected the index of all adipose tissues and the most efficient reduction was obtained with combined drugs (GSOR) (Fig. 1C–F). We reported the effect of OR, GSSE and combined treatments on fat index (Fig. 1G) and liver index (Fig. 1I). In SD groups, the total fat index was significantly decreased by the double treatment (Fig. 1G). In HFD groups, this index was significantly decreased by OR and GSOR. The liver index was partially corrected by the three treatments (Fig. 1I).

### Analysis of blood serum parameters

**Fig. 2:**
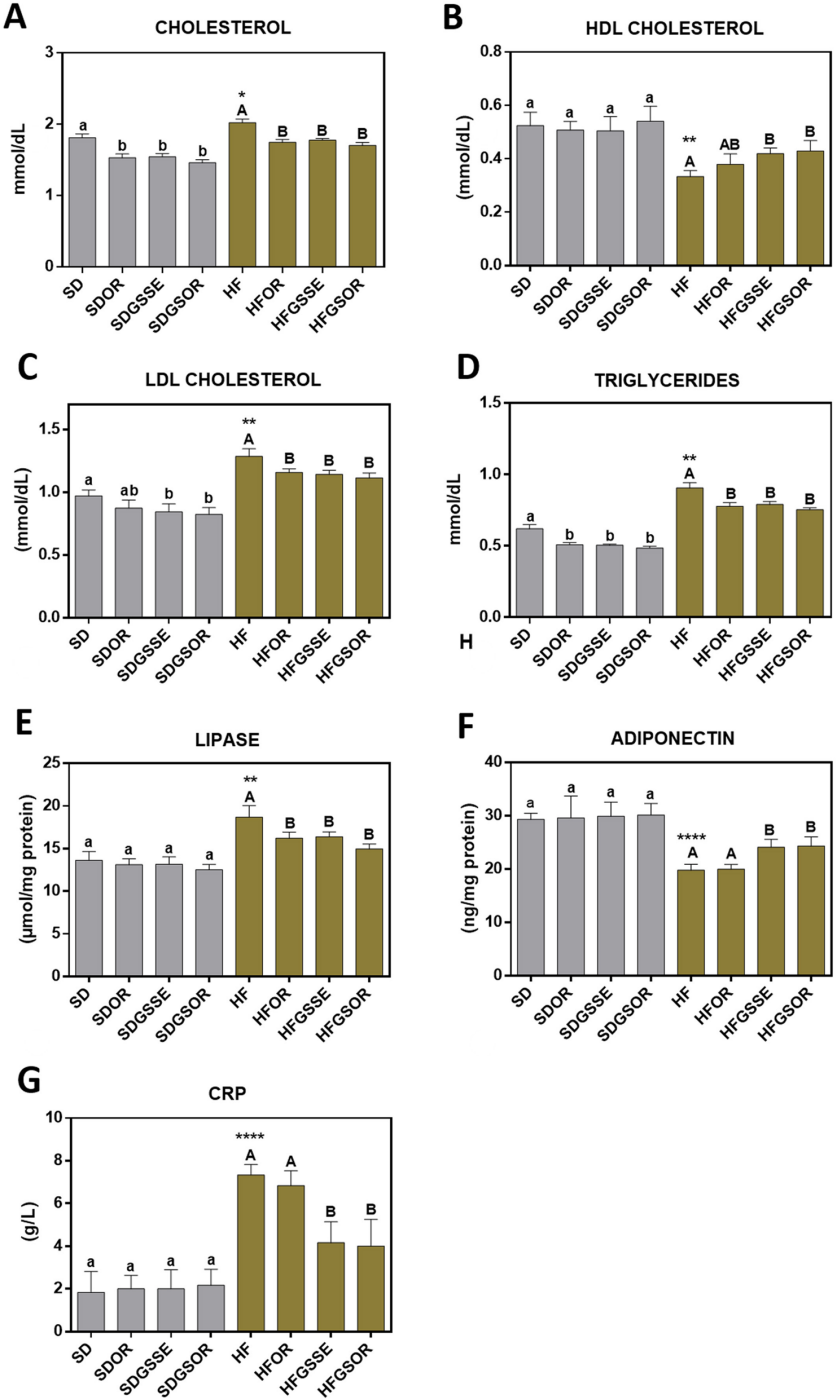
Effect of OR, GSSE, and GSOR on SD (grey) and HF diet (brown) serum cholesterol (**A**), serum HDL (**B**), serum LDL (**C**), serum Triglycerides (**D**), serum lipase (**E**), serum adiponectin (**F**), serum CRP (**G**). Data are expressed as mean ± SEM (n = 6). On top of each bar, lowercase and capital letters indicate significant differences analyzed by two-way ANOVA followed by Tukey’s test (P < 0.05) for vs.SD and vs. HF diet, respectively. The asterisk represents the significant difference analyzed by parametric t-test (P < 0.05) for SD vs. HF. **** indicates 0.001 < P-value < 0.01; *** indicates 0.001 < P-value < 0.01; ** indicates 0.001 < P-value < 0.01; * 0.01 < P-value < 0.05

The total cholesterol (TC), HDL, LDL, triglycerides (TG), adiponectin, lipase and C-reactive protein (CRP) levels in serum in each group were summarized in Fig. 2A–G. HFD increased TC, LDL, TG, lipase, and CRP levels, and decreased HDL (good cholesterol) and adiponectin levels. These results are in accord with the extensive literature studies on obesity. GSSE and GSOR significantly corrected all serum parameters tested, with higher efficiency of the double treatment. Contrariwise, the orlistat alone did not improve HDL, adiponectin and CRP levels. We also observed some significant modifications in serum parameters with the SD treatments. The GSSE and the GSOR decreased TC, LDL, and TG. However, OR only decreased TC and TG levels. None of the treatments modified the HDL, lipase, adiponectin and CRP levels.

### Microbiota analysis

The Illumina MiSeq platform analyzed GM changes associated with HFD-induced obesity and GSSE, OR or both drugs. 1,489,131 sequencing reads for the fecal microbiota were analyzed using GALAXY and SHAMAN and assigned to OTUs.

### Bacterial diversity across treatments in fecal contents at six months

Alpha diversity, representing the microbial diversity within each sample, was analyzed based on the genus richness. The Shannon and Simpson and inverse Simpson indexes were calculated at the same genus level.

**Fig. 3:**
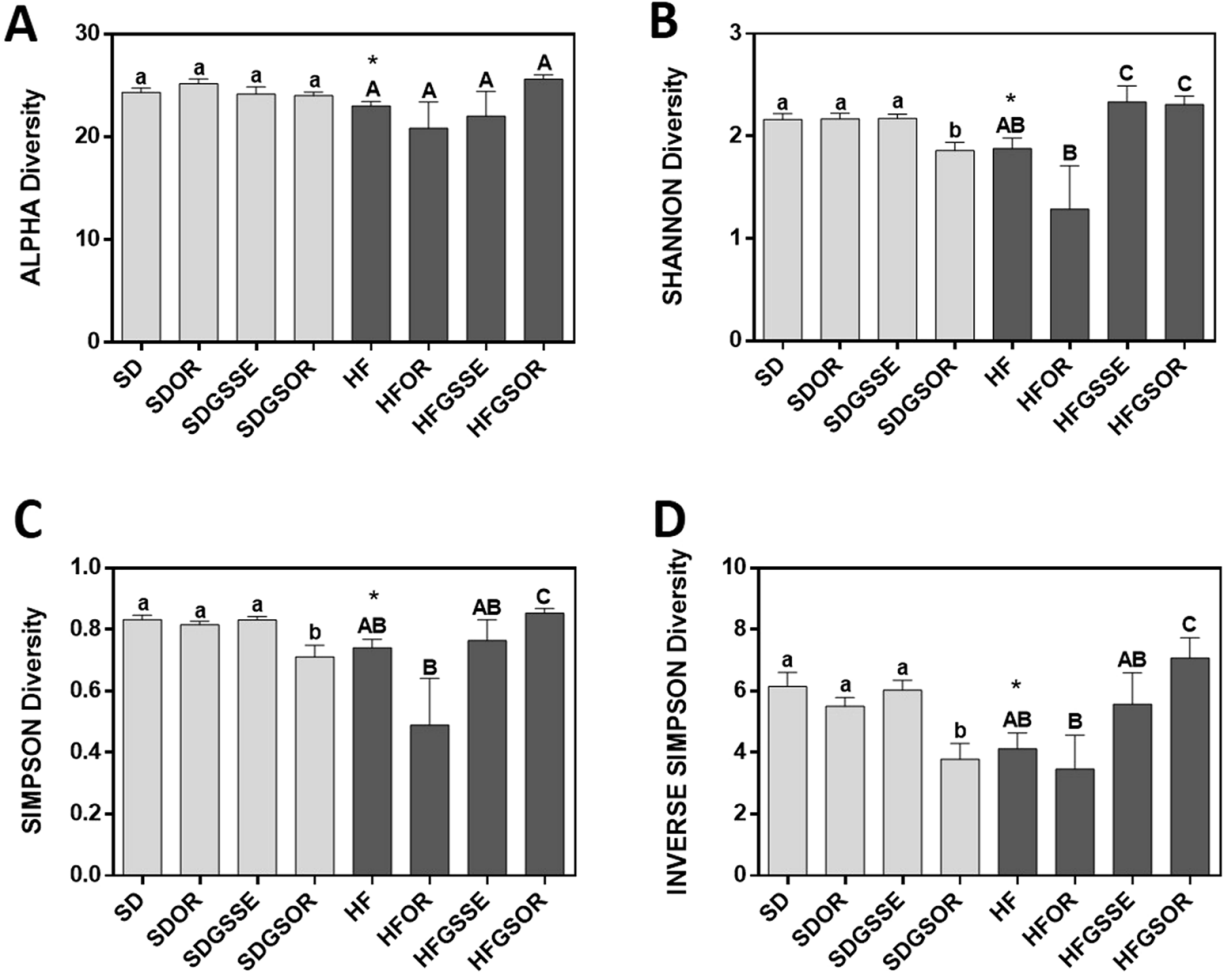
Effect of OR, GSSE, and GSOR on SD (light grey) and HFD (dark grey) induced alpha diversity (**A**), Shannon diversity (**B**), Simpson diversity (**C**), and inverse Simpson diversity (**D**). Data are expressed as mean ± SEM (n = 6). On top of each bar, lowercase and capital letters indicate significant differences analyzed by two-way ANOVA followed by Tukey’s test (P < 0.05). The asterisk represents the significant difference analyzed by parametric t-test (P < 0.05) for SD vs. HF. **** indicates 0.001 < P-value < 0.01; *** indicates 0.001 < P-value < 0.01; ** indicates 0.001 < P-value < 0.01; * 0.01 < P-value < 0.05

The observed genus number was lower in HFD than in the SD group, but no significant difference was observed between the four HFD groups and the four SD groups (Fig. 3A). At the genus level, the Shannon diversity index, the Simpson, and the inverse Simpson indexes were significantly lower in the HF diet group than in the SD group (p < 0.05) (Fig. 3B–D). Considering the Shannon index, GSSE and GSOR treatments restored the loss of GM diversity observed in the HF diet group (Fig. 3B). In contrast, only the GSOR treatment restored the Simpson and the inverse Simpson indexes (Fig. 3C, D). Comparing SD and SD treated groups, these three indexes were lower only in the GSOR treatment indicating that orlistat combined with GSSE can reduce GM diversity in rats fed with an SD (Fig. 3B–D).

### Bacterial composition and differential abundance across treatments in fecal contents at six months

**Fig. 4:**
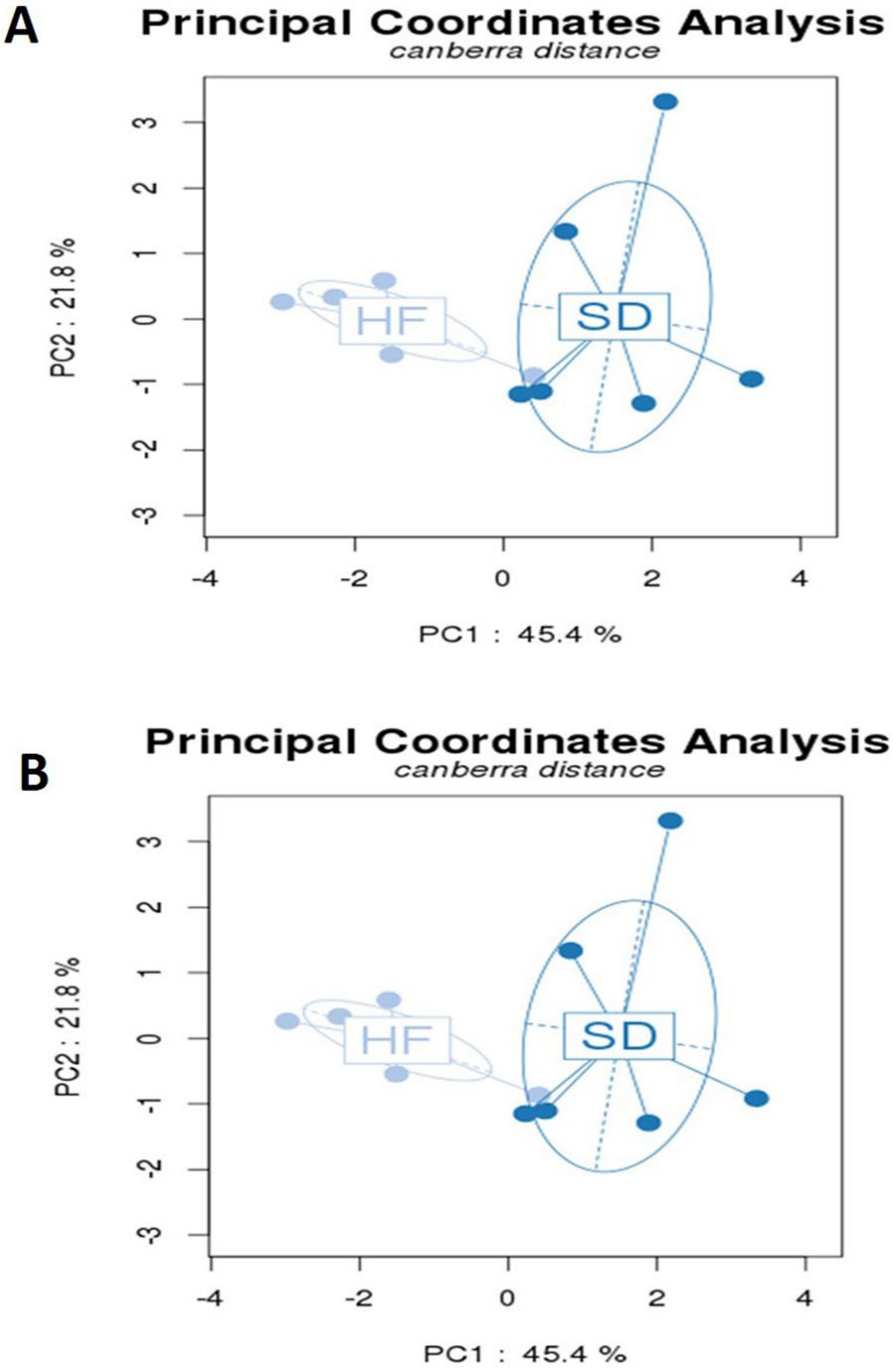
At the end of the treatment, the diet induces significant modifications in the fecal microbiota of SD (light blue) and HF diet (dark blue) groups. The pattern of microbiota clustering according to the diagnosis as assessed by principal coordinate analysis on Canberra distance: **A** at phylum level (P = 0.005) and **B** at family level (P = 0.009)

To determine the structural changes in the GM, we compared the relative abundance of the predominant taxa identified from the four HF diets and four SD groups at the end of the treatments (6 months of diet administration). Significant differences (p < 0.05) existed in the composition of the GM at all taxonomic levels among the groups. First, we studied the effect of the HF diet vs. the control diet (SD). The microbiota of the SD was significantly different compared to the HFD, as shown in the Principal Composant Analysis (PCoA) of the phylum (P = 0.005) and families (P = 0.009) (Fig. 4A, B).

**Fig. 5:**
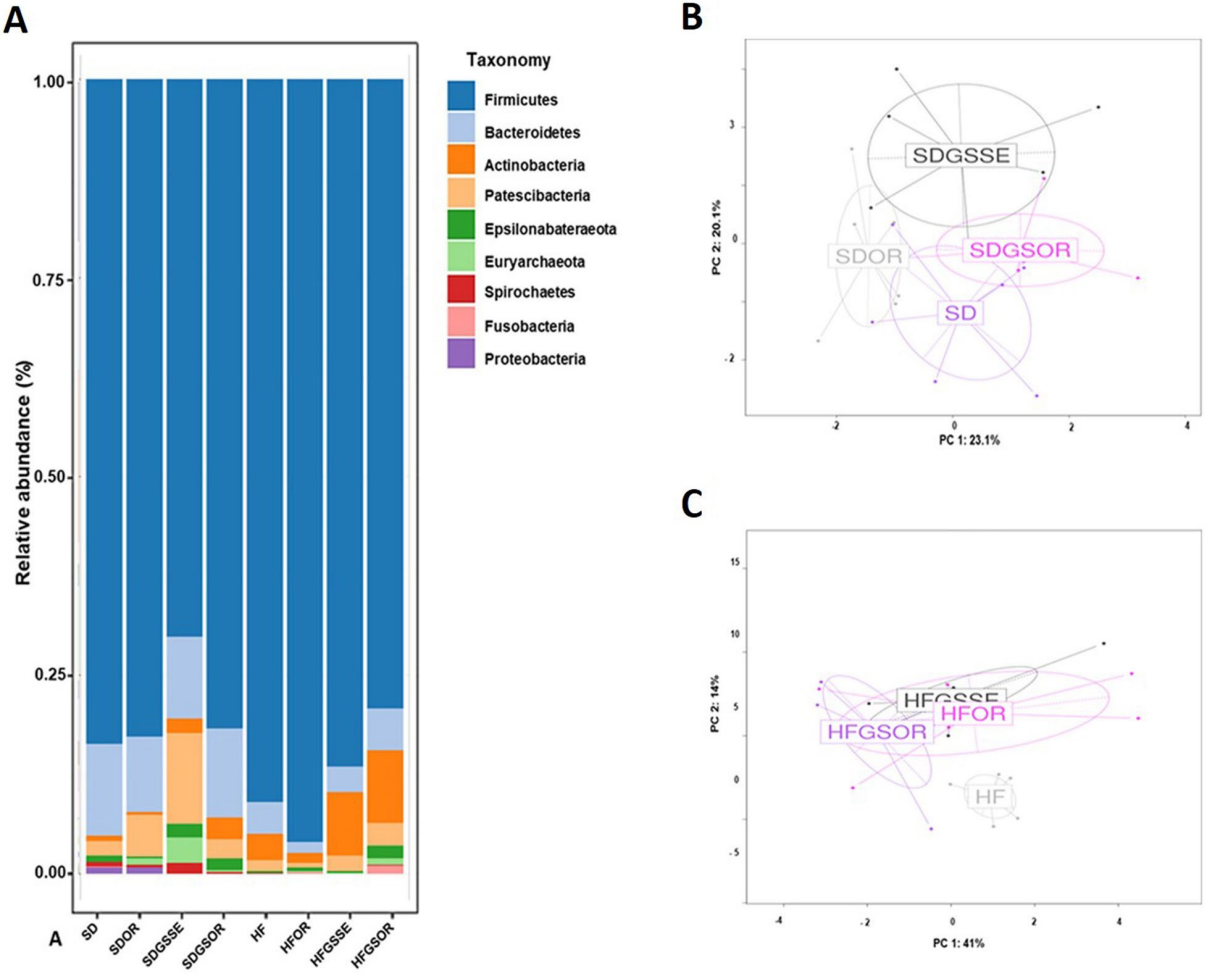
Taxonomic composition of the GM under different types of treatments at the phylum level: **A** Barplot of the proportion of different taxa in the different conditions. **B** Principal coordinate analysis on Canberra distance for SD groups (P = 0.001) and **C** for HF diet groups (P = 0.017)

Significant differences at phylum level are represented in the heatmap of Fig. 7A. The relative abundance of phylum, families, and genus is represented in the additional information (Additional file 2: Table S2). Firmicutes and Bacteroidetes were the most represented phyla but we did not find significant differences between the SD and HF groups (Fig. 5A, 7A). However, the HF diet increased the Actinobacteria phylum (0.7% vs 3.3%), represented principally by *Bifidobacterium* (0% vs 3%), and in minority by *Coriobacteriaceae*, and lowered the Proteobacteria (0.8% vs 0%) (a minority phylum, represented by *Escherichia* genus) (Fig. 5A, 6A). No significant differences were observed in the other phyla.

**Fig. 6:**
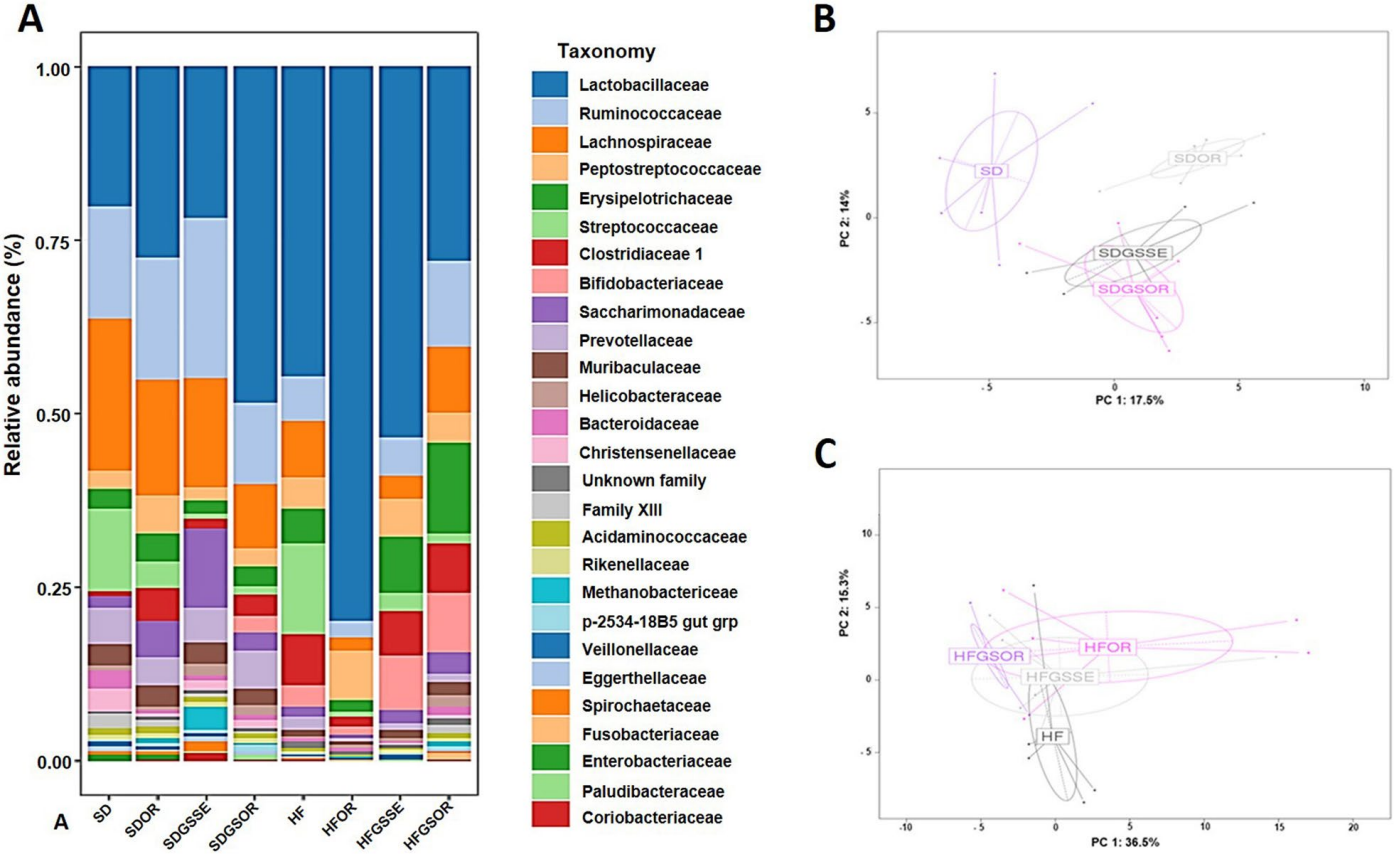
Taxonomic composition of the GM under different types of treatments at the family level: **A** Barplot of the proportion of different taxa in the different conditions. **B** Principal coordinate analysis on Canberra distance for SD groups (P = 0.07) and **C** for HF diet groups (P = 0.037)

**Fig. 7:**
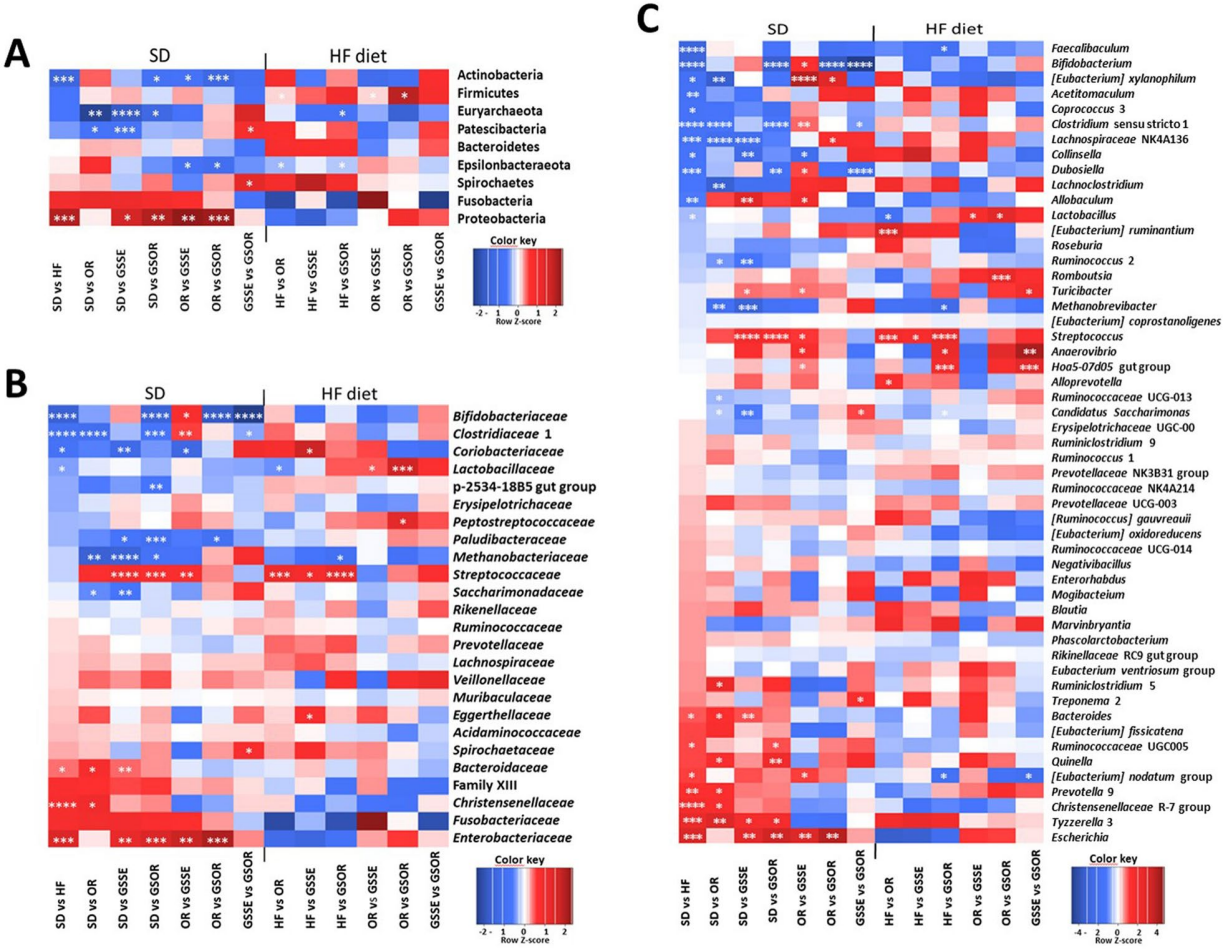
Heatmap of log2 fold changes obtained for the different contrasts. **** indicates 0.001 < P-value < 0.01; *** indicates 0.001 < P-value < 0.01; ** indicates 0.001 < P-value < 0.01; * 0.01 < P-value < 0.05

Significant differences at the family level are represented in the heat map of Fig. 7B, and their relative abundances are on Additional file 2: Table S2. *Lactobacillaceae* (the major family represented by the *Lactobacillus* genus) increased in HFD compared to SD (20.3% vs 44.6%). Of the same, *Erysipelotrichaceae* were increased (2.8% vs 5.1%) in HF diet as well as *Clostridiaceae* 1 (0.6% vs 7.4%), *Bifidobacteriaceae* (0% vs 3%) and the minority family *Coriobacteriaceae* (0% vs 0.2%) (Fig. 6A, 7B). In addition, HF diet had decreased *Christensenellaceae,* (represented by *Christensenellaceae* R7 group) (3.3% vs 0.1%), *Bacteroidaceae* (represented by *Bacteroides* genus) (2.5% vs 0.4%) as well as *Enterobacteriaceae* (represented by *Escherichia* genus) (0.8% vs 0%) (Fig. 6A, 7B).

Significant differences at the genus level are represented in the heat map of Fig. 7C, and their relative abundances in (Additional file 2: Table S2). *Lactobacillus*, the majority genus, was increased in HFD (20.3% vs 44.7%) as well as *Bifidobacterium* (0% vs 3%), *Clostridium* SS1 (1% vs 7.4%), *Dubosiella* (0% vs 1.4%), *Lachnospiraceae* NK4A136 group (0% vs 1.6%), and six minority genera (< 1%): *Allobaculum*, *Faecalibaculum, Collinsella, Eubacterium xylanophilum* group, *Coprococcus* 3 and *Acetitomaculum*.

HFD decreased *Ruminococcaceae* UCG-005 (7% vs 1%), *Christensenellaceae* R7 group (3.3% vs 0%), *Bacteroides* (2.5% vs 0.4%), *Prevotella* 9 (1.5% vs 0.1%), and *Tyzzerella* (a member of *Lachnospiraceae* family) (1.1% vs 0%) and two minority genera (< 1%): *Escherichia* and *Eubacterium nodatum* group.

**Fig. 8:**
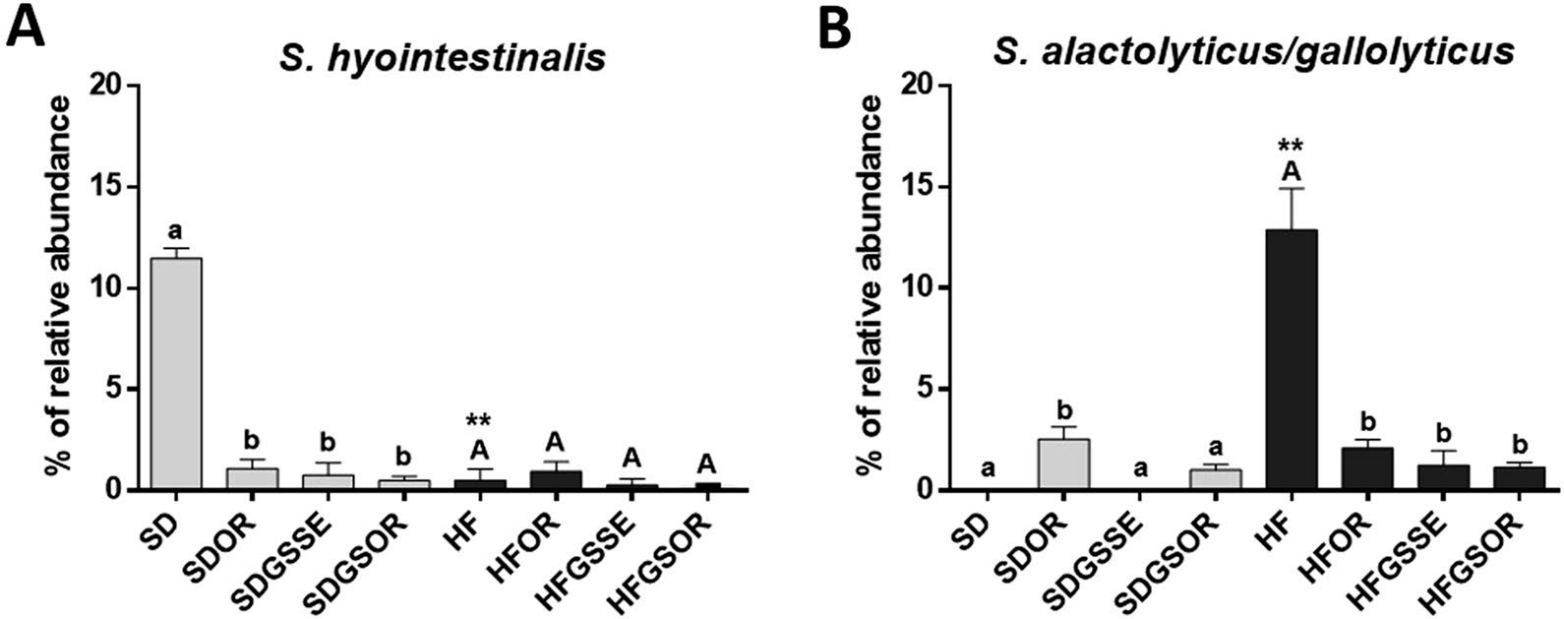
Barplot of the proportion of the two *Streptococcus* species in the different diets. **A** Barplot of the *S. hyointestinalis* (grey) in the different conditions. **B** Barplot of the *S. alactolyticus/ gallolyticus* in the different conditions. On top of each bar, lowercase and capital letters indicate significant differences analyzed by two-way ANOVA followed by Tukey’s test (P < 0.05) for vs. SD and vs. HF diet, respectively. The asterisk represents the significant difference analyzed by a t-test (P < 0.05) for SD vs. HF

Microbiota populations of the four HF diet groups were significantly separated as represented in PCoA plots (Fig. 5C and 6C), indicating that the GM was changed after the three treatments. The most significant modification concerned the *Streptococcus* genus. This genus was highly decreased with the three treatments. *Streptococcus* represented 13% of the total GM in the HF diet, decreasing to 0.6%, 2.6% and 1.3% in OR, GSSE and SGOR treatments, respectively (Fig. 7C, 8A, B). A deep blasting of the 16S rRNA sequences corresponding to the *Streptococcus* genus was realized. In the HF diet group, *S. alactolyticus/gallolyticus* (belonging to the *Streptococcus bovis* group) was identified as the major species. In the SD group *Streptococcus hyointestinalis* was identified as the major species (Fig. 8A, B). This species is included in the miscellaneous group of *Streptococcus* (group VII) [36]. Therefore, we observed a substituting of one *Streptococcus* species with another between the rats subjected to HF and SD diets. Partial 16S rRNA sequences of these two *Streptococcus* species were 96% homologous.

In addition, GSSE decreased *Coriobacteriaceae* (0.2% vs 0%) and *Eggerthellaceae* (0.2% vs 0.1%) (Actinobacteria increased in the HF diet). However, orlistat increased Firmicutes (91% vs 96%) and *Lactobacillus* (44.7% vs 80%). This great increase of *Lactobacillus* could explain the decreased alpha-diversity in the microbiota observed with the OR treatment. Additionally, OR decreased the minor represented genera *Alloprevotella* and *Eubacterium ruminantium* group. The combined treatment increased *Faecalibaculum* (0.2% vs 2%), *Candidatus Saccharimonas* (1% vs 3%), *Methanobrevibacter* (0% vs 0.8%) and *Eubacterium nodatum* group (0.1% vs 0.6%). More, GSOR decreased two-minority genus, *Anaerovibrio* and hoa5-07 d05 (Fig. 7A–C and Additional file 2: Table S2).

Likewise, we studied the effect of the three treatments in rats fed with the SD (SD groups), and, therefore not obese. GM modifications at all taxonomic levels were observed with each treatment. Since the starting GM should have already been different at the start of the treatments (non-obese vs obese rats), the expected changes could be completed in the same direction as those found in the rats fed with the HF diet. The PCoA plots (Fig. 5B, 6B) revealed that the GM of the four SD groups is very distant, even more than between the HF diet groups. Similarly to HF treatments, the *Streptococcaceae* family (represented by the *Streptococcus* genus) decreased in all SD treatments. The *Streptococcus* genus represented 12% of the total GM in SD feed rats, decreasing to 4%, 0.7%, and 1.3% in OR, GSSE, and SGOR treatment groups, respectively (Fig. 7C, 8A, B). As represented in Fig. 8A, *Streptococcus hyointestinalis* was the major species. In addition, all treatments significantly increased the Euryarchaeota phylum (represented by the *Methanobrevibacter* genus)*, *especially GSSE. Moreover, the Patescibacteria phylum (represented by the *Saccharimonas* genus) was increased in OR and GSSE, the Actinobacteria were increased in GSOR, and Gammaproteobacteria (minority phyla, < 0.8%, represented by *Enterobacteriaceae*) were decreased in GSSE and GSOR (Fig. 5A, 7A). On the contrary, Firmicutes, and Bacteroidetes*,* the most represented phyla, were not significantly modified with treatments.

Variations between treatments observed at the genus level were greater compared to those observed in obese rats. *Clostridium* ss1 (1% vs 4.7%)*,* the *Lachnospiraceae* NK4A136 group (0% vs 3.4%)*, Ruminococcus* 2 (0.4% vs 2%), and *Ruminococcaceae* UCG-013 (0.6% vs 2%) were increased with orlistat. Moreover, *Christensenellaceae* R-7 group (3.3% vs 0.5%)*, Bacteroides* (2.5% vs 0.6%), *Prevotella* 9 (1.5% vs 0.3%) and *Tyzzerella* 3 (1.1% vs 0%), were decreased in OR group, together with the modifications of five minor genera (Fig. 7C). The GSSE treatment increased the *Lachnospiraceae* NK4A136 group (0% vs 3.3%)*, Collinsella* (0% vs 1%), and *Ruminococcus* 2 (0.4% vs 2.7%)*,* and decreased *Bacteroides* (2.5% vs 0.7%), *Tyzzerella* 3 (1.1% vs 0.1%), and *Turicibacter* (2.3% vs 1%) and modified three minor genera. Finally, the combined treatment increased *Bifidobacterium* (0% vs 2.4%) *Clostridium* s.s1 (1% vs 3%) and *Dubosiella* (0% vs 1.7%), decreased *Ruminococcaceae* UCG-005 (7% vs 0.8%) and *Tyzzerella* 3 (1.1% vs 0.1%), and modified two minor genera (Fig. 7C).

## Discussion

Obesity and metabolic syndrome result from multifactorial issues, including host genome, lifestyle, diet, and GM [20, 21]. The restoration of GM dysbiosis is crucial in treating obesity by influencing energy metabolism and the immune system. Most animal models studying the impact of polyphenols in obesity used preventive models (treatments were administered at the beginning of the fattening). Here, in a curative model of obesity, we investigated the effect of three treatments on physiological parameters and GM modulation. According to our results, GSSE decreases blood cholesterol and attenuates lipid accumulation in HF diet administered rats [7]. Only a few studies have focused on the effect of the combined polyphenol-orlistat treatment. Our results are in agreement with the anti-obesity and GM modulation effects observed with grape and blueberry polyphenols and orlistat treatments [7, 28, 37].

The relationship between GM and the effects of orlistat and grape polyphenols on obesity has not yet been completely established. Previous studies demonstrated that an HF diet generally decreases microbiota diversity indexes [37–39]. In our study, the diversity (Shannon index) significantly decreased in HF diet, but orlistat did not improve GM diversity. In contrast, GSSE supplementation improved this diversity and restored the loss of diversity observed after orlistat treatment. These results are in agreement with those signaling the capacity of polyphenols to improve GM diversity [37, 39].

A large study in humans identified a solid and consistent taxonomic signature of obesity to provide potential targets for obesity prevention and treatment [40]. In obese individuals, abundance of *Streptococcaceae* and *Lactobacillaceae* families increased, whereas *Christensenellaceae*, *Clostridiaceae*, and *Dehalobacteriaceae* abundance decreased. Our results suggested that the most critical GM modification caused by the three treatments in the HF diet groups was the sharp decrease in the relative abundance of *Streptococcaceae* family and *Streptococcus* genus. Faecal bacteria belonging to the *Streptococcus* genus were associated with the development of various metabolic disorders and obesity [40–42], as knee pain in osteoarthritis [43] and even multiple sclerosis [44]*.*

We were able to identify the specific streptococci retrieved in HF diet fed rats such as *S. alactolyticus/gallolyticus*, which belongs to the *S. bovis* complex group [45].These streptococci coloning both humans and animals can be opportunistic pathogens inducing various diseases and inflammations. *S. gallolyticus* is associated mainly with early adenomas and may thus constitute an early marker for colorectal cancer screening. Interestingly, a genomic analysis of various *S. gallolyticus* and related species demonstrated the presence of bacteriocin operons, like those of gallocins, which contribute to their gut colonization, killing closely related gut commensals, and thus enabling better colon colonization [46]. In this context, GSSE, orlistat or combined treatment could represent an excellent alternative to displace *Streptococcus* gut colonization.

In a previous study, we demonstrated significant anti-inflammatory effects of combined GSSE-orlistat treatment in HF diet rats [7], leading us to hypothesize a possible correlation between the significant presence of *S*. *alactolyticus/gallolyticus* (13% of relative abundance) and the pro-inflammatory phenotype. However, other studies must be carried out to confirm this hypothesis. Recently, Gu et al. [47] reported that COVID-19 patients presented a reduced bacterial diversity and an increased relative abundance of opportunistic pathogens in faeces, such as *Streptococcus* and *Rothia*, and that these taxa were positively associated with the CRP inflammatory score.

Despite differences between the human and murine gut microbiotas [48], we observed in the present study some interesting microbiota similarities between the obese rats and those described in COVID-19 patients [49, 50]. For instance, an increase in *Streptococcus*, *Coriobacteriaceae* (*Collinsella*) and *Clostridium*, as well as a decrease in *Bacteroides* and in bacterial groups producing short-chain fatty acids (SCFA). GSSE treatment was able to counterbalance some of these modifications. Interestingly, polyphenols were able to impact positively COVID-19 infection, as stated by various authors [51, 52].

In addition to *Streptococcus*, GSSE significantly reduced *Eggerthellaceae* and *Coriobacteriaceae,* this latter being able to affect the physiology of human and mouse hosts [53, 54] as their number harbored a positive correlation with hepatic triglycerides or with plasma non-HDL cholesterol levels [55]. *Coriobacteriaceae* family was also shown to increase significantly in stressed mice [56] and has been linked with schizophrenia [57] and to patients suffering from bipolar disorders [58]. On the other hand, a study on humans identified *Eggerthella lenta* as a specie linked with the occurrence of T2D [59].

Furthermore, the double treatment (GSOR) was able to produce the most significant number of GM modifications in obese rats, reducing potential pathobionts such as hoa5-07d05 (*Rikenellaceae* group) [60], *Turicibacter *[61] and the lipid catabolizer *Anaerovibrio*, present in T2D rats [62]. Conversely, GSOR increased (1) *Methanobrevibacter*, a methanogen bacterium previously associated with studies bearing on lean animals [63], (2) *Eubacterium,* a potentially beneficial bacterium that forms part of the human gut microbiome core [64] and (3) *Faecalibaculum*, able to produce butyrate and lactic acid as major metabolic end products, which has been related with anti-obesity effects; this suggests their potential role as probiotic for preventive and therapeutic applications [38, 65, 66]. Nevertheless some controversy still exist because of the putative negative role of *Faecalibaculum* in metabolic diseases [67].

Furthermore, we noticed an increase of *Lactobacillus* in HFD fed animals treated with OR, that could be linked to the well-established ability of Lactobacilli to proliferate when fatty acids are abundant [68], as recently described for *L. rhamnosus* GG, that consumes fatty acids which lead to the reduction in intestinal fatty acid absorption and alleviate body fat accumulation [69, 70]. Thus the extensive increase in intestinal lipid level following OR treatment could be at the basis of higher number of *Lactobacillus*.

It is well known that approximately 90% of dietary polyphenols reaches the colon, where they modulate microbiota composition and function leading to host benefits [71]. In this context, it seems essential to discuss the impact of GSSE in GM of non-obese rats fed with the SD. GSSE increased beneficial bacteria such as *Methanobrevibacter*, able to synthesize vitamin B and break down several toxins such as TMAO [72]. GSSE also increased *Ruminococcus* 2 and *Lachnospiraceae* NK4A136 respectively a SCFA producer and a potential probiotic [73]. Interestingly apart from its clear ability to reduce *Streptococcus* spp, GSSE also reduced some pathobionts such as *Enterobacteriaceae* (*Escherichia* genus), *Allobaculum, Turicibacter* and *Tyzzerella*3. *Allobaculum* was shown to be positively correlated to intestinal inflammation and leaky gut [74], *Tyzzerella* with increased cardiovascular disease risk [75], and *Turicibacter* with rheumatoid arthritis and constipation in humans, and was detected in abundance in tumor-bearing mice [76–78].

## Conclusion

Obese rats presented altered physiological parameters, a loss of GM diversity, and more abundance of some potential gut pathobionts compared to lean animals. Combining GSSE and orlistat (GSOR) appeared to be the most efficient treatment. It improves body weight, serum lipid parameters, fat accumulation and gut microbiota diversity, increasing beneficial microbes and reducing potential pathobionts. GSSE could be proposed as an excellent complement to OR treatment of obesity and could also find relevant applications in other pathologies involving GM alterations in the lean animal.

## Methods

### Reagents and diets

GSSE was obtained from a grape cultivar (Carignan) of *Vitis vinifera*. Seeds and skin were processed, dried, and grinded separately till a fine powder was obtained and dissolved in 10% ethanol at (a 50/50) ratio. Extraction of polyphenols as well as quantitative and qualitative composition, were conducted as described[33]. Orlistat (OR) ((S)-2-formylamino-4-methyl-pentanoic acid (S)-1-[[(2S, 3S)-3-hexyl-4-oxo-2oxetanyl] methyl]-dodecyl ester) was obtained from Pharmalpa (France) and dissolved in 10% ethanol (v/v). Standard diet (SD) was obtained from ALMAS (Tunisia), and SD was supplemented with 20% animal origin fat to obtain high-fat diet (HFD) [79]. Rats were daily treated by oro-gastric gavage with GSSE (4 g/kg bw), OR (2 mg/kg bw) or both drugs.

### Animal experimentation

Forty-eight male Wistar rats of 12 weeks old were obtained from Pasteur Institute Tunis, and housed in a controlled environment (3 rats/cage) in agreement with the Local Ethics Committee of Carthage University that approved the experimental protocol and with NIH (National Research Council) guidelines. After one week of adaptation, rats were allocated into two groups fed either SD or HFD for three months (diets compositions Additional file 2). Then each group was divided into four subgroups (n = 6) that were treated for three other months with GSSE (SDGSSE and HFGSSE), OR (SDOR and HFOR), or combined drugs (GSSE + OR) (SDGSOR and HFGSOR). Control SD and HF received 10% ethanol in water as vehicle, and all the treatments were given by oro-gastric gavage.

### Physiological analysis

During the entire period of treatment, animals were daily observed and followed weekly for weight loss. At the end of the treatment rats were anesthetized with urethane (40 mg/mL), sacrificed, their blood collected into heparinized tubes, and processed for plasma biomarkers determination. Liver and adipose tissues as perirenal adipose tissue (PAT), retroperitoneal adipose tissue (RAT), mesenteric adipose tissue (MAT), and perigonadal adipose tissue (PGAT) were collected, weighed and organ index expressed using the following formula: organ index = [organ weight (g)]/[body weight]. Total cholesterol, HDL-cholesterol, LDL-cholesterol, and triglycerides were performed using commercially available kits from Biomaghreb (Tunisia). Determination of lipase activity was made according to Humbert et al. [80]. Adiponectin was analyzed via Assay Max rat adiponectin ELISA Kit (ASSAYPRO®, MO, USA). C-reactive protein (CRP) was determined using a Konelab Clinical Chemistry Analyzer (Thermo Clinical Labsystems, Espoo, Finland).

### Stool sampling and fecal DNA extraction

One day before the end of the protocol, stools were collected in sterile tubes and stored at -80 °C. Around 100 mg of faeces were accurately weighed and homogenized in Tris–EDTA buffer (Tris 0.1 mM, pH 8; EDTA 1 M (Sigma); 1 mL of buffer for 200 mg of faeces). Lysozyme (1:100, 300 mg/mL (Sigma)) was added to the mixture, and samples were incubated at 37 °C for one hour. Then, 200 µL of the mixture was used in the DNA isolation kit, NucleoSpin® Soil kit (Macherey–Nagel) according to manufacturer’s instructions. The NanoDrop One (Thermo Fisher Scientific, USA) was used to determine DNA concentration and purity.

### Microbiota analysis by Illumina sequencing

The V3–V4 region of the 16S rRNA gene was amplified using forward primer 338F (5′ACTCCTACGGGAGGCAGCA-3′) and reverse primer 806R (5′-GGACTACHVGGGTWTCTAAT-3′) for Illumina library construction. The PCR mixture was prepared using the kit MP Taq DNA Pol (USA),g 10 ng of DNA, 10 μM of each primer, and PCR grade water to a final volume of 50 μl. PCR cycling conditions consisted of an initial denaturation of 5 min at 95 °C, 30 cycles of 30 s at 95 °C, annealing at 52 °C for 30 s, and extension at 72 °C for 45 s, and a final extension at 72 °C for 2 min. The length and concentration of the PCR product were detected by 1% agarose gel electrophoresis. DNA amplicons were sequenced using the Illumina MiSeq platform (Genotoul, France).

The effective sequences were assessed by GALAXY FROGS to discard low-quality sequences and amplicons with wrong size. Paired-end joined sequences were grouped into operational taxonomic units (OTUs) and clustered using Swarm as previously described (aggregation parameter d = 1 + d = 3) [34]. After removing chimera with VSEARCH, OTUs presenting more than 0.005% of the total number of sequences were kept. 124 OTUs were classified using the reference data base silva138 with pintail quality100.

All the OTUs were blasted with NCBI blast for checking the taxonomy. The mean number of reads per sample was 31,023 (min: 18,486 − max: 58,588) 87,78% of sequences with an amplicon size between 420 and 480 were kept 44,6% of the clusters and 22,8% of the sequences were removed after the VSEARCH chimera step. Removing clusters with abundances lower than 0.005% eliminated 49,6% of the sequences and 99,9% of the OTUs. Samples were grouped according to treatments, and normalized using the DESeq2 method. Alpha diversity, Shannon, Simpson and inverse Simpson indices were calculated at genus level to characterize this diversity, using SHAMAN [35]. Significance in abundance variation between samples by heatmap, PCoA, and other statistical analyses were performed with SHAMAN [35].

### Statistical analysis

Data were compared by two-way analysis of variance (ANOVA) followed by Tukey’s multiple comparison tests. Significance among SD and HF groups was analyzed by unpaired Student’s t-test. Results were expressed as the mean ± SEM. A P value less than 0.05 was considered significant.

## Supplementary Information

**Additional file 1**. Composition of the SD and the HFD.

**Additional file 2**. Relative relative abundance of the GM under different types of treatments at Phylum, Family and Genus levels.

## Data Availability

All data generated or analyzed in this study are included in this article.
