# Supplementary material for: Grape seed and skin extract, a potential prebiotic with anti-obesity effect through gut microbiota modulation
Source: Gut Pathog. 2022 Jul 6;14:30. doi: 10.1186/s13099-022-00505-0 (PMC9258160; doi:10.1186/s13099-022-00505-0)
Supplement: Supplementary file 1 — Additional file 1. Composition of the SD and the HFD. [file 13099_2022_505_MOESM1_ESM.pdf]

Parameter	SD	HFD
**Lipid %(w/w)**	3	28
**Carbohydrate %(w/w)**	40	32
**protein %(w/w)**	14	12
**Fiber %(w/w)**	7,5	6
**Moisture %(w/w)**	14	12
**Ash %(w/w)**	9	7,2
**Methionin %(w/w)**	0,46	0,37
**Cystein %(w/w)**	0,34	0,27
**Threonine %(w/w)**	0,782	0,62
**Tryptophane %(w/w)**	0,25	0,2
**Copper (mg/kg)**	10	8
**Manganese (mg/kg)**	50	40
**Zinc (mg/kg)**	50	40
**Iron (mg/kg)**	40	32
**Calcium (mg/kg)**	1,2	0,94
**Magnesium (mg/kg)**	0,05	0,04
**Phosphorus (mg/kg)**	0,5	0,4
**Selenium (mg/kg)**	0,1	0,08
**Iodide (mg/kg)**	0,7	0,56
**Cobalt (mg/kg)**	0,1	0,08
