# Supplementary material for: Grape seed and skin extract, a potential prebiotic with anti-obesity effect through gut microbiota modulation
Source: Gut Pathog. 2022 Jul 6;14:30. doi: 10.1186/s13099-022-00505-0 (PMC9258160; doi:10.1186/s13099-022-00505-0)
Supplement: Supplementary file 2 — Additional file 2. Relative relative abundance of the GM under different types of treatments at Phylum, Family and Genus levels. [file 13099_2022_505_MOESM2_ESM.pdf]

PHYLUM	SD	SDOR	SDGSSE	SDGSOR	HF	FHOR	HFGSSE	HFGSOR
Firmicutes	83,6	82,8	70,1	81,7	91	96	86,5	79,2
Bacteroidetes	11,6	9,4	10,3	11,2	4	1,4	3,2	5,3
Patescibacteria	1,8	5,2	11,4	2,5	1,4	0,6	1,9	2,9
Actinobacteria	0,7	0,4	1,9	2,7	3,3	1,2	8	9,1
Euryarchaeota	0	0,7	3,2	0,3	0	0,1	0,1	0,8
Epsilonbacteraeota	0,8	0,3	1,7	1,4	0,2	0,5	0,3	1,6
Spirochaetes	0,6	0,4	1,4	0,2	0,1	0	0	0,1
Proteobacteria	0,8	0,8	0	0	0	0	0	0
Fusobacteria	0,1	0	0	0	0	0,2	0	1
TOTAL	100	100	100	100	100	100	100	100

FAMILY	SD	SDOR	SDGSSE	SDGSOR	HF	FHOR	HFGSSE	HFGSOR
Lactobacillaceae	20,3	27,5	21,8	48,3	44,6	79,7	53,4	27,9
Ruminococcaceae	16	17,6	23,1	11,8	6,4	2,4	5,4	12,4
Lachnospiraceae	21,8	16,6	15,6	9,2	8,1	1,9	3,4	9,7
Streptococcaceae	11,9	3,8	0,7	1,3	13,1	0,6	2,6	1,3
Erysipelotrichaceae	2,8	4	2	2,9	5,1	1,8	8,1	13,1
Saccharimonadaceae	1,8	5,2	11,4	2,5	1,4	0,6	1,9	2,9
Bifidobacteriaceae	0	0	0	2,4	3	1,1	8	8,7
Clostridiaceae 1	0,6	4,7	1,4	3	7,4	1,5	6,3	7,2
Peptostreptococcaceae	2,8	5,5	1,9	2,5	4,6	7,2	5,5	4,1
Prevotellaceae	5,1	4	4,9	5,5	2	0,4	1	1,2
Christensenellaceae	3,3	0,5	1,5	1	0,1	0	0,3	0,5
Muribaculaceae	3,1	3,2	3,1	2,4	1	0,5	1,2	2,1
Methanobacteriaceae	0	0,7	3,2	0,3	0	0,1	0,1	0,8
Bacteroidaceae	2,5	0,6	0,7	0,9	0,4	0,3	0,3	1,2
Family XIII	2,2	0,8	0,4	0,3	0,1	0,1	0,1	1,2
Helicobacteraceae	0,8	0,3	1,7	1,4	0,2	0,5	0,3	1,6
Spirochaetaceae	0,6	0,4	1,4	0,2	0,1	0	0	0,1
Coriobacteriaceae	0	0,1	1,1	0,1	0,2	0	0	0,1
Acidaminococcaceae	1	0,9	0,8	0,5	0,3	0,1	0,2	0,9
Rikenellaceae	0,6	0,9	0,7	0,8	0,3	0,1	0,4	0,3
p-2534-18B5 gut grp	0,2	0,5	0,6	0,9	0,3	0,1	0,2	0,4
Enterobacteriaceae	0,8	0,8	0	0	0	0	0	0
Eggerthellaceae	0,7	0,3	0,8	0,3	0,2	0,1	0,1	0,3
Veillonellaceae	0,7	0,3	0,3	0,1	0,3	0,1	0,7	0,1

Paludibacteraceae	0	0,1	0,3	0,7	0,1	0	0,1	0,1
Fusobacteriaceae	0,1	0	0	0	0	0,2	0	1
TOTAL	99,6	99,3	99,4	99,3	99,3	99,4	99,6	99,3

**GENUS**	**SD**	**SDOR**	**SDGSSE**	**SDGSOR**	**HF**	**FHOR**	**HFGSSE**	**HFGSOR**
Lactobacillus	20,3	27,4	21,8	48,3	44,7	80	53,5	27,9
Unknown genus	12	4,3	11,4	8,8	4,6	1,5	3,2	8,6
Streptococcus	11,9	4	0,7	1,3	13	0,6	2,6	1,3
Blautia	12,6	6,6	2,7	5,2	3,4	0	1	2,7
Candidatus Saccharimonas	1,8	5,2	11,5	2,5	1	0,5	2	3
Bifidobacterium	0	0	0	2,4	3	0,6	8	9
Romboutsia	3,5	7,5	2,5	3	5	8	6	4,1
Clostridium sensu stricto 1	1	4,7	1,4	3	7,4	1,5	6,3	7,2
Ruminococcaceae UCG-005	7	5,4	6,6	0,8	1	1	1,2	2,6
Dubosiella	0	0,6	0	1,7	1,4	0,2	1,5	6,4
Ruminococcaceae UCG-014	3	3	6	3	1	0,5	1	2,4
Turicibacter	2,3	2,7	1	1	2,7	1	4,5	1,4
Prevotellaceae NK3B31 grp	2	3	4	4	1	0,8	0,5	0,6
Lachnospiraceae NK4A136 grp	0	3,4	3,3	0,5	1,6	1	0,5	2
Christensenellaceae R-7 grp	3,3	0,5	1,5	1	0	0	0,3	0,5
Methanobrevibacter	0	0,7	3,2	0,3	0	0,1	0,1	0,8
Allobaculum	0	0	0,1	0	0,7	0,3	1,5	3,1
Ruminococcus 2	0,4	2	2,7	1,3	0,6	0,5	0,3	1,1
Bacteroides	2,5	0,6	0,7	1	0,4	0,3	0,3	1,2
[Eubacterium] coprostanoligenes grp	1,2	2	2	1,3	1,3	0	1	2,4
Faecalibaculum	0	0	0	0	0,2	0,2	0,5	2,2
Ruminococcus 1	1,5	0,7	1,4	1,3	0,8	0,2	0,5	1
Ruminococcaceae UCG-013	0,6	2	1,5	1,1	0,1	0,2	0,5	0,5
Prevotella 9	1,5	0,3	0,6	0,5	0,1	0,1	0,2	0,1
Treponema 2	0,6	0,4	1,4	0,2	0,1	0	0	0,1
Ruminococcaceae NK4A214 grp	0,7	1,1	1,2	1	0,3	0,2	0,4	1
Tyzzerella 3	1,1	0	0,1	0,1	0	0	0	0
Phascolarctobacterium	1	1	0,8	0,5	0,3	0	0,1	1
Prevotellaceae UCG-003	1	0,3	0,4	0,3	0,3	0,1	0,2	0,2
Erysipelotrichaceae UCG-003	0,2	0,7	1	0,4	0,1	0	0,1	0,1
Collinsella	0	0,1	1	0,1	0,2	0	0	0,1
[Ruminococcus] gauvreauii grp	0,6	0,9	0,5	0,4	0,2	0	0,1	0,5

[Eubacterium] ventriosum grp	0,3	0,5	0,9	0,3	0,1	0	0	0,1
Lachnoclostridium	0	0,9	0,1	0,1	0	0	0	0
Marvinbryantia	0,1	0,8	0,9	0,4	0	0	0	0
Escherichia	0,8	0,8	0	0	0	0	0	0
[Eubacterium] xylanophilum grp	0	0,8	0	0	0,1	0	0,1	0,5
[Eubacterium] ruminantium grp	0,4	0,7	0,6	0,1	0,8	0	0,1	0,1
[Eubacterium] nodatum grp	0,5	0,7	0,1	0,2	0,1	0	0	0,6
Ruminiclostridium 5	0,7	0	0,4	0,1	0,2	0,1	0,1	0,2
Rikenellaceae RC9 gut grp	0,4	0,5	0,6	0,5	0,1	0,1	0,1	0,3
[Eubacterium] fissicatena	0,6	0,1	0,5	0,3	0,1	0	0,1	0,3
Alloprevotella	0,5	0,6	0,2	0,4	0,4	0	0,2	0,3
Enterorhabdus	0,3	0,2	0,6	0,1	0,1	0	0	0,1
Coprococcus 3	0	0,2	0	0,1	0,2	0,1	0,1	0,6
Anaerovibrio	0,2	0,3	0	0,1	0,2	0,1	0,6	0
Quinella	0,5	0,2	0,2	0	0,1	0	0	0,1
Ruminiclostridium 9	0,4	0,3	0,4	0,2	0,2	0,1	0,2	0,3
Roseburia	0,1	0,2	0,4	0,3	0,2	0	0,1	0,3
hoa5-07d05 gut grp	0,2	0,4	0,1	0,3	0,2	0	0,3	0
Mogibacterium	0,1	0,1	0,3	0	0	0	0	0,2
Negativibacillus	0,1	0,2	0,1	0,1	0	0	0	0,3
[Eubacterium] oxidoreducens grp	0,1	0,1	0,2	0,1	0	0	0	0,3
Acetitomaculum	0	0,1	0,1	0	0,2	0,1	0	0,2
TOTAL	99,9	99,8	99,7	100	99,8	100	99,9	99,9
